# A pre-averaged pseudo nearest neighbor classifier

**DOI:** 10.7717/peerj-cs.2247

**Published:** 2024-08-13

**Authors:** Dapeng Li

**Affiliations:** School of Software Engineering, Jinling Institute of Technology, Nanjing, China

**Keywords:** Pre-averaged, Pseudo nearest neighbors, Small-size samples

## Abstract

The k-nearest neighbor algorithm is a powerful classification method. However, its classification performance will be affected in small-size samples with existing outliers. To address this issue, a pre-averaged pseudo nearest neighbor classifier (PAPNN) is proposed to improve classification performance. In the PAPNN rule, the pre-averaged categorical vectors are calculated by taking the average of any two points of the training sets in each class. Then, k-pseudo nearest neighbors are chosen from the preprocessed vectors of every class to determine the category of a query point. The pre-averaged vectors can reduce the negative impact of outliers to some degree. Extensive experiments are conducted on nineteen numerical real data sets and three high dimensional real data sets by comparing PAPNN to other twelve classification methods. The experimental results demonstrate that the proposed PAPNN rule is effective for classification tasks in the case of small-size samples with existing outliers.

## Introduction

Supervised learning is an important field of machine learning. Random forest (RF) (Breiman, 2001), support vector machine (SVM) (Cortes & Vapnik, 1995), and k-nearest neighbor (kNN) (Cover & Hart, 1967) are common supervised learning methods. The kNN algorithm is a simple and effective machine learning method. It was first proposed for classification (Cover & Hart, 1967) in 1967. This algorithm assigns a test sample to the class represented by most of the k-nearest neighbors in the training set. In the kNN rule, the asymptotic classification performance can be achieved using the Bayes method under sufficient conditions (Li, Chen & Chen, 2008). It is one of the top 10 classification algorithms in the data mining field (Wu et al., 2008). Hence, kNN has been widely studied. Many kNN-based classification methods have been proposed (Li, Chen & Chen, 2008; Pan, Wang & Ku, 2017; Memis, Enginoǧlu & Erkan, 2022b; Zeng, Yang & Zhao, 2009a; Chai et al., 2021; Memis, Enginoǧlu & Erkan, 2022a; Memis, 2023; Keller, Gray & Givens, 1985; Mullick, Datta & Das, 2018; Memis, Enginoǧlu & Erkan, 2022c; Erkan, 2021; Kumbure, Luukka & Collan, 2018). Only one parameter, $k$, can be selected for kNN rule. If $k$ is too small or large, the classifier is sensitive to noise points and outliers (Gou et al., 2019c). Hence, the classification accuracy of kNN is easily affected by outliers, especially in the case of small size training sets (Zhang, 2022).

To deal with the issue, some effective methods have been reported to reduce the negative impact of outliers. A local mean-based nearest neighbor classifier (Mitani & Hamamoto, 2006) (LMkNN) has been proposed. The categorical mean vectors in LMkNN rule can somewhat counteract the influence of outliers. The pattern of a test point is determined according to the weighted distance sum of *k*-nearest points chosen from each class in the pseudo nearest neighbor (Zeng, Yang & Zhao, 2009b) (PNN) rule, based on the distance weighted $k$-nearest neighbor (WkNN) rule (Dudani, 1976). The classification performance is better than kNN in small-size training sample case with outliers.

As an extension of both LMkNN and PNN, a local mean-based pseudo nearest neighbor classifier (LMPNN) was proposed (Gou et al., 2014). The category of a test point is predicted according to the weighted distance sum between the *k*-pseudo nearest neighbors and the test point in LMPNN classifier rule. The classification accuracy is higher than kNN, LMkNN, and PNN. Based on the ideas of PNN, LMkNN, and LMPNN, some derived methods have been reported (Pan, Wang & Ku, 2017; Gou et al., 2022, 2019b; Ma et al., 2022; Gou et al., 2019a; Pan et al., 2021; Gou et al., 2019c; Zhang et al., 2019; Gou et al., 2012; Gong et al., 2023; Xu et al., 2013) to improve classification performance.

Although several kNN-based classification methods have been proposed, how to further reduce the negative impact of outliers is still an open issue. Based on the above methods, pre-averaged pseudo-pre-averaged vectors from training samples are used. Then, the test sample is classified according to the distances between the first $k$-pseudo local nearest vectors and the test sample. Unlike the PNN method, the k-nearest neighbors are selected from the pre-averaged vectors in each class to determine the query point pattern in the PAPNN rule. The selected *k*-pseudo local nearest neighbors can further reduce the negative impact of outliers to some degree. The effectiveness and superiority of the proposed method are verified by experiments on numerical real sets and high dimensional real data sets.

The key work in this article is summarized as follows: 1. A pre-averaged pseudo nearest neighbor classifier is proposed. In this method, the categorical mean vectors are obtained by taking an average of two random points from training samples in each class. Then, the first $k$-pseudo local nearest neighbors from pre-averaged vectors of each class are selected in the PAPNN rule. The selected $k$-pseudo nearest neighbors are somewhat less sensitive to outliers. Hence, compared to conventional kNN, PAPNN can improve classification performance in the case of small-size samples with existing outliers. 2. Experiments are conducted to verify the effectiveness and superiority of PAPNN.

The remainder of this article is structured as follows. In the “Related Work” is briefly summarized. In the “Proposed PAPNN Method” is introduced. “Experiment” presents extensive experiments on numerical data sets and high dimensional data sets. Finally, a brief conclusion is drawn in “Conclusion”.

## Related work

In this section, some related typical kNN-based classifiers are briefly reviewed. Portions of this text were previously published as part of a preprint (Li, 2024).

### PNN classifier

PNN rule utilizes $k$-nearest neighbors from each class to determine a query point category. More class information can be captured compared to the traditional kNN. Hence, PNN can obtain better performance than kNN. The algorithm is described as follows:

(1) Calculate the distance between the training sample ${y_{ji}}$ and a test sample $y$ from each class by euclidean distance:
(1) $$d\left( {y,{y_{ji}}} \right) = \sqrt {{{\left( {y - {y_{ji}}} \right)}^T}\left( {y - {y_{ji}}} \right)} ,$$
where ${y_{ji}}$ represents the $i$th sample of class ${\omega _j}$, $j = 1,2,...,M$, *M* represents the number of classes. Then, the first $k$-nearest distances $d_1^j$, $d_2^j$,…, $d_k^j$ for class ${\omega _j}$ can be obtained by sorting $d(y,{y_{ji}})$ in an ascending order.

(2) Assume that $y_j^{PNN}$ denotes the pseudo neighbor from class ${\omega _j}$. The distance $d\left( {y_j^{PNN},y} \right)$ can be written as:
(2) $$d(y_j^{PNN},y) = {w_1}d_1^j + {w_2}d_2^j + ... + {w_k}d_k^j,$$
where ${w_1},{w_2},...,{w_k}$ can be defined as ${w_i} = 1/i$ according to PNN rule.

(3) The test sample $y$ is classified into the class with the minimum distance of $d\left( {y_j^{PNN},y} \right)$ among all classes.

### LMkNN classifier

As an extension of kNN, LMkNN (Mitani & Hamamoto, 2006) is a robust and simple nonparametric classifier. It is less sensitive to $k$ than kNN. Point average method in LMkNN rule can reduce the influence of outliers in some degree, especially in the case of small-size training samples with existing outliers.

A query sample $y \in {R^D}$ in a *D* dimensional feature space is classified into class ${\omega _i}$ by the following steps:

*Step 1*. Calculate the euclidean distances between the test sample $y$ and training samples from each class ${\omega _i}$. Then, *k*-nearest neighbors $y_1^i$, $y_2^i$,…, $y_k^i$ in each class can be obtained, where $i$ denotes a class number, according to the ascending order of the calculated distance.

*Step 2*. Calculate the local mean vector ${y_i}$ for every class:

(3) $${y_i} = {1 \over k}\sum_{j = 1}^k {y_j^i} .$$

*Step 3*. Calculate the distance between the local mean vector ${y_i}$ and the query point $y$ for class ${\omega _i}$. Then, the pattern of the query sample $y$ is finally classified into the class with the minimum distance between each categorical local mean vector and $y$ among all classes.

### LMPNN classifier

In LMPNN rule, multi local-mean based pseudo nearest neighbors in each class are utilized to determine a query sample pattern. Compared to PNN and LMkNN, LMPNN can obtain better classification accuracy rate in the case of small-size training samples with outliers. LMPNN rule is carried out as follows:

*Step 1*. Compute the distances between training samples in each class $j$ and a query point $y$:

(4) $$d(y,{y_{ji}}) = \sqrt {{{(y - {y_{ji}})}^T}(y - {y_{ji}})} ,$$

where ${y_{ji}}$ represents the $i$th training sample in class $j$. Then, the k-nearest neighbors ( ${y_{1j}}$, ${y_{2j}}$,…, ${y_{kj}}$) of $y$ for each class can be found by sorting the distances.

*Step 2*. Compute $k$ local mean vectors for each class:

(5) $$\overline {{x_{ij}}} = {1 \over i}\sum_{f = 1}^i {{y_{fi}}} ,j = 1,2,...,k,$$

where $\overline {{x_{ij}}}$ denotes the $i$th local mean vector of class $j$.

*Step 3*. Let $\overline {x_j^{PNN}}$ represents the categorical pseudo nearest neighbor of $y$ from class $j$. The weighted distances sum between the local mean-based pseudo nearest neighbor $\overline {x_j^{PNN}}$ and the unclasified point $y$ among each class are computed as:

(6) $$d(y,\overline {x_j^{PNN}} ) = d\left( {y,\overline {{x_{1j}}} } \right) + \left( {{1 \over 2}d\left( {y,\overline {{x_{2j}}} } \right)} \right) + ... + \left( {{1 \over k}d\left( {y,\overline {{x_{kj}}} } \right)} \right).$$

*Step 4*. The pattern of the query point $y$ is classified into the class $w$ when the euclidean distance $d\left( {y,\overline {x_w^{PNN}} } \right)$ is minimum among all classes. Note that LMPNN, PNN, LMkNN is equivalent with $k = 1$.

## The proposed papnn classifier

In this section, a pre-averaged pseudo nearest neighbor classifier based on kNN is introduced. The purpose of PAPNN algorithm is to improve classification performance in the case of small-size training samples with existing outliers. Portions of this text were previously published as part of a preprint (Li, 2024).

### The basic idea

In kNN rule, the classification accuracy is easily affected by outliers, especially in the case of small-size training points. The selection of the value $k$ significantly influences classification performance. A small $k$ may result in outliers being chosen as nearest neighbors, thereby compromising classification performance (Gou et al., 2019c). Conversely, in certain cases, the accuracy may be degraded with a large $k$ value, as it incorporates numerous points from other classes among the $k$-nearest neighbors (Gou et al., 2019c).

The methods, such as LMkNN, PNN, and LPMNN, have been reported to improve classification performance in the case of small-size training samples with existing outliers. LMkNN can overcome the negative impact of the existing outliers to some degree. However, the same number of nearest neighbors for each class and the same weight coefficients for the nearest neighbors may affect classification effect (Gou et al., 2019c; Pan, Wang & Ku, 2017). In the PNN rule, larger weight coefficients are assigned to closer neighbors, which reduces the negative impact of outliers to some extent. Nevertheless, outliers can still compromise classification performance when the value of $k$ is excessively large. LMPNN employs multiple pseudo local mean vectors derived from the $k$-nearest neighbors of each class to categorize a query point. Compared to LMkNN (Gou et al., 2014), LMPNN captures more class-specific information, thereby resulting in superior classification performance.

Based on the ideas of the aforesaid article, a pre-averaged based pseudo nearest neighbor classifier is introduced to reduce the adverse effects of outliers in the case of small-size training samples with existing outliers.

### PAPNN rule

Let $S = ({y_j},{c_i})_{j = 1}^N$ be a training set with *N* training samples for *M* classes, where $i = 1,2,...,M$, ${y_j} \in {R^d}$, $d$ is the feature dimension. ${S_i} = ({y_{ij}},{c_i})_{j = 1}^{{n_i}}$ stands for the training sample set of class ${c_i}$, where ${n_i}$ is the number of the training points in class ${c_i}$. In the proposed PAPNN rule, the class label of an unclassified point $y$ can be assigned by the following steps:

1. Calculate the pre-averaged vectors in each class by the following formula:

(7) $$\eqalign{{x_{iq}}& = ({y_{ij}} + {y_{in}})/2,j = 0,1,...,({n_i} - 1),n = (j + 1),...,{n_i},q \\&= 1,2,...,{n_i}({n_i} - 1)/2,{n_i} \; > \; 1,}$$

where ${x_{iq}}$ denotes the $q$th averaged vector of class ${c_i}$. Note that ${x_{iq}} = {y_{i1}}$ with ${n_i} = 1$.

2. Compute the distances between the pre-averaged vectors in each class ${c_i}$ and $y$:

(8) $$d(y,{x_{iq}}) = \sqrt {{{(y - {x_{iq}})}^T}(y - {x_{iq}})} .$$

Then, the smallest $k$ distances $\left( {d\left( {y,\overline {{x_{i1}}} } \right),d\left( {y,\overline {{x_{i2}}} } \right),...,d\left( {y,\overline {{x_{ik}}} } \right)} \right)$ can be obtained by sorting $d(y,{x_{iq}})$ in an ascending order.

3. Let $y_i^{PAPNN}$ denotes the categorical pseudo nearest neighbor of $y$ from class ${c_i}$. The weighted distance sum between the $y_i^{PAPNN}$ and $y$ among all classes are computed as:

(9) $$d\left( {y,y_i^{PAPNN}} \right) = \sum_{j = 1}^k {{1 \over j}} d\left( {y,\overline {{x_{ij}}} } \right).$$

4. Classify $y$ as the class ${c_j}$ with the minimal distance:

(10) $${c_j} = argmind\left( {y,y_j^{PAPNN}} \right).$$

It should be noted that PAPNN, LMkNN, PNN, and LMPNN are equivalent with $k = 1$. As mentioned above, the proposed PAPNN method is shown in Algorithm 1.

**Algorithm 1:** Outline of the proposed algorithm.

**Require:**
$y$: an unclassified point, $k$: the number of nearest neighbors.
$Y = y1,y2, ...,y_n$: $n$ training points for $m$ classes.
$Y_j = y_{1 j},\;y_{2 j},\;...,y_{n j}$: represents a training subset from the class ${c_j}$ with ${n_j}$
training samples.
*C* = { ${c_1}$, ${c_2}$,…, ${c_M}$}: the set of *M* classes.
**Ensure:**
The class label of the unclassified point $y$.
** Step 1:** Calculate the pre-averaged vectors for each class with $k \;> \; 1$.
**for** $i$ = 1 to *M* **do**
$q = 0;$
**for** $j$ = 0 to $({n_j} - 1)$ **do**
**for** $n$ = j+1 to ${n_j}$ **do**
$x_{iq}$ = $({y_{ij}} + {y_{in}})/2$, $q + +$;
**end for**
** end for**
** end for**
Note that ${x_{i1}} = {y_{j1}}$ with $k = 1$.
**Step 2:** Calculate the distances between ${n_j}({n_j} - 1)/2$ mean vectors in each
class ${c_j}$ and $y$.
**for** $q$ = 1 to $({n_j}({n_j} - 1)/2)$ **do**
$d(y,{x_{jq}}) = \sqrt {{{(y - {x_{jq}})}^T}(y - {x_{jq}})}$
**end for**
Then, find first $k$ pseudo local nearest points of class ${c_j}$, denoted as $y_j^{PAPNN}$ =
{ $\overline {{x_{j1}}}$, $\overline {{x_{j2}}}$,…, $\overline {{x_{jk}}}$}.
**Step 3:** Compute the weighted distance sum between the first $k$ pseudo nearest
neighbors and the query point $y$ among each class.
**for** $j$ = 1 to *M* **do**
**for** $i$ = 1 to $k$ **do**
$d\left( {y,y_j^{PAPNN}} \right)$ += ${1 \over i}d\left( {y,\overline {{x_{ji}}} } \right)$
**end for**
**end for**
** Step 4**: The query sample $y$ is classified into the category with the minimal
distance.
$c_j^y$ = $argmind\left( {y,y_j^{PAPNN}} \right)$.

## Experiments

To validate the classification performance of PAPNN rule, the extensive experiments on numerical real data sets and three high dimensional data sets are carried out to evaluate the performance of PAPNN method.

### Data sets

In this subsection, the information of the selected data sets used in the experiments is presented.

The nineteen numerical real world data sets are taken from the UCI Machine Learning Repository (Bache & Lichman, 2013) and the KEEL Repository (Alcalá-Fdez et al., 2011), which are Vehicle, Balance, Blood, Bupa, Ionosphere, Pima-indians, Parkinsons, Hill, Haberman-survival, Musk-1, Sonar, Wine, Cardiotocography, QSAR, Band, Pima, Wine(keel), Mammographic and Steel, respectively. Among these nineteen real-world data sets, The numbers of total samples, attributes, classes of each data set, sources, and whether it is imbalanced are also listed in the first $19$ rows of Table 1. From the first $19$ rows Table 1, it can be seen that the data sets have different characteristics in numbers of attributes, samples, and classes. The numbers of all samples in the selected data sets are mostly small. These data sets can be used to verify the proposed method classification performance in the small training sample size cases.

**Table 1:** The data sets used in the experiments.

Data	Samples	Attributes	Classes	Source	Imbalance
Vehicle	$846$	$18$	$4$	UCI	Yes
Balance	$625$	$4$	$3$	UCI	Yes
Blood	$748$	$4$	$2$	UCI	Yes
Bupa	$345$	$6$	$2$	UCI	Yes
Ionosphere	$351$	$34$	$2$	UCI	Yes
Pima-Indians	$768$	$8$	$2$	UCI	Yes
Parkinsons	$195$	$22$	$2$	UCI	Yes
Hill	$1,\!210$	$100$	$2$	UCI	No
Haberman-survival	$306$	$3$	$2$	UCI	Yes
Musk-1	$476$	$166$	$2$	UCI	Yes
Sonar	$208$	$60$	$2$	UCI	Yes
Wine	$178$	$13$	$3$	UCI	Yes
Cardiotocography	$2,\!126$	$21$	$10$	UCI	Yes
QSAR	$1,\!055$	$41$	$2$	UCI	Yes
Band	$365$	$19$	$2$	KEEL	Yes
Pima	$768$	$8$	$2$	KEEL	Yes
Wine(keel)	$178$	$13$	$3$	KEEL	Yes
Mammographic	$830$	$5$	$2$	KEEL	Yes
Steel	$1,\!941$	$27$	$7$	UCI	Yes
AndrogenReceptor	$1,\!687$	$1,\!024$	$2$	UCI	Yes
Semeion	$1,\!593$	$256$	$2$	UCI	No
CNAE-9	$1,\!080$	$857$	$9$	UCI	Yes

### Experiments on the real data sets

Cross-validation is a common method to evaluate the performance of machine learning models. Among the various folds of cross-validation, five-fold and $10$-fold cross-validation are commonly used (Erkan, 2021), (Kumbure, Luukka & Collan, 2018). Compared to five-fold cross-validation, $10$-fold cross-validation can obtain more reliable performance results. Therefore, $10$-fold cross validation is adopted in the experiments. Afterward, to ensure the reliability of performance results, $10$ runs are carried out, and the average results are obtained for a specific value of the nearest neighbor $k$. The value of the nearest neighbor parameter $k$ are varied from $1$ to $20$ with a step of $1$. The classification results are determined by averaging the classification results of $k = 1,2,...,20$. Experiments were performed by utilizing Python 3.7.3 and a computer with Intel(R) Core(TM) CPU i5-10210U @ 1.60GHz and 8 GB RAM.

Table 2 shows the comparison results between PAPNN and the other methods, *i.e*. kNN (Cover & Hart, 1967), WkNN (Dudani, 1976), PNN (Zeng, Yang & Zhao, 2009b), LMkNN (Mitani & Hamamoto, 2006), LMPNN (Gou et al., 2014), fuzzy kNN (FkNN) (Keller, Gray & Givens, 1985), eigenvalue classification method (EigenClass) (Erkan, 2021), generalized mean distance-based k-nearest neighbor classifier (GMDkNN) (Gou et al., 2019a), fuzzy k-nearest neighbor classifier based on the Bonferroni mean (BMFkNN) (Kumbure, Luukka & Collan, 2018), fuzzy parameterized fuzzy soft k-nearest neighbor classifier (FPFS-kNN) (Memis, Enginoǧlu & Erkan, 2022b), SVM (Cortes & Vapnik, 1995), and random forests (RF) (Breiman, 2001). The settings of the compared algorithms are summarised in Table 3.

**Table 2:** Comparative results for the data sets.

Data	Methods	Accuracy $\pm$ SD	Precision $\pm$ SD	Recall $\pm$ SD	F1-score $\pm$ SD
Vehicle	kNN	0.7028 $\pm$ 0.0020	0.7016 $\pm$ 0.0022	0.7028 $\pm$ 0.0020	0.6898 $\pm$ 0.0012
	WkNN	0.7146 $\pm$ 0.0016	0.7164 $\pm$ 0.0017	0.7146 $\pm$ 0.0016	0.7080 $\pm$ 0.0011
	PNN	0.7152 $\pm$ 0.0012	0.7150 $\pm$ 0.0016	0.7152 $\pm$ 0.0012	0.7042 $\pm$ 0.0015
	LMkNN	0.7324 $\pm$ 0.0015	0.7436 $\pm$ 0.0014	0.7324 $\pm$ 0.0015	0.7313 $\pm$ 0.0015
	LMPNN	0.7324 $\pm$ 0.0015	0.7436 $\pm$ 0.0014	0.7324 $\pm$ 0.0015	0.7313 $\pm$ 0.0015
	PAPNN	**0.7777 $\pm$ 0.0007**	**0.7851 $\pm$ 0.0006**	**0.7777 $\pm$ 0.0007**	**0.7761 $\pm$ 0.0009**
	FkNN	0.7103 $\pm$ 0.0017	0.7106 $\pm$ 0.0021	0.7103 $\pm$ 0.0017	0.6990 $\pm$ 0.0019
	EigenClass	0.6782 $\pm$ 0.0016	0.6744 $\pm$ 0.0016	0.6782 $\pm$ 0.0016	0.6660 $\pm$ 0.0010
	GMDkNN	0.7315 $\pm$ 0.0012	0.7397 $\pm$ 0.0011	0.7315 $\pm$ 0.0012	0.7280 $\pm$ 0.0012
	BMFkNN	0.7312 $\pm$ 0.0014	0.7426 $\pm$ 0.0014	0.7312 $\pm$ 0.0014	0.7304 $\pm$ 0.0009
	FPFS-kNN	0.6585 $\pm$ 0.0022	0.6498 $\pm$ 0.0029	0.6585 $\pm$ 0.0022	0.6397 $\pm$ 0.0012
	SVM	0.7245 $\pm$ 0.0008	0.7210 $\pm$ 0.0013	0.7245 $\pm$ 0.0008	0.7117 $\pm$ 0.0011
	RF	0.7517 $\pm$ 0.0002	0.7539 $\pm$ 0.0003	0.7517 $\pm$ 0.0002	0.7457 $\pm$ 0.0002
Balance	kNN	0.8705 $\pm$ 0.0016	0.8282 $\pm$ 0.0031	0.8705 $\pm$ 0.0016	0.8446 $\pm$ 0.0003
	WkNN	0.8278 $\pm$ 0.0022	0.8034 $\pm$ 0.0038	0.8278 $\pm$ 0.0022	0.8144 $\pm$ 0.0008
	PNN	0.8932 $\pm$ 0.0010	0.8330 $\pm$ 0.0029	0.8932 $\pm$ 0.0010	0.8594 $\pm$ 0.0002
	LMkNN	0.9000 $\pm$ 0.0009	0.8903 $\pm$ 0.0019	0.9000 $\pm$ 0.0009	0.8853 $\pm$ 0.0074
	LMPNN	0.9000 $\pm$ 0.0009	0.8903 $\pm$ 0.0019	0.9000 $\pm$ 0.0009	0.8853 $\pm$ 0.0074
	PAPNN	**0.9456 $\pm$ 0.0006**	**0.9469 $\pm$ 0.0006**	**0.9456 $\pm$ 0.0006**	**0.9422 $\pm$ 0.0043**
	FkNN	0.8721 $\pm$ 0.0017	0.8232 $\pm$ 0.0037	0.8721 $\pm$ 0.0017	0.8458 $\pm$ 0.0001
	EigenClass	0.8647 $\pm$ 0.0014	0.8091 $\pm$ 0.0031	0.8647 $\pm$ 0.0014	0.8343 $\pm$ 0.0002
	GMDkNN	0.8737 $\pm$ 0.0007	0.8348 $\pm$ 0.0028	0.8737 $\pm$ 0.0007	0.8503 $\pm$ 0.0006
	BMFkNN	0.8399 $\pm$ 0.0010	0.8482 $\pm$ 0.0022	0.8399 $\pm$ 0.0010	0.8408 $\pm$ 0.0052
	FPFS-kNN	0.8890 $\pm$ 0.0010	0.8283 $\pm$ 0.0028	0.8890 $\pm$ 0.0010	0.8565 $\pm$ 0.0003
	SVM	0.8849 $\pm$ 0.0015	0.8195 $\pm$ 0.0033	0.8849 $\pm$ 0.0015	0.8493 $\pm$ 0.0023
	RF	0.8193 $\pm$ 0.0014	0.8281 $\pm$ 0.0031	0.8193 $\pm$ 0.0014	0.8225 $\pm$ 0.0020
Blood	kNN	0.7634 $\pm$ 0.0022	0.7450 $\pm$ 0.0037	0.7634 $\pm$ 0.0022	0.7413 $\pm$ 0.0040
	WkNN	0.7414 $\pm$ 0.0018	0.7229 $\pm$ 0.0034	0.7414 $\pm$ 0.0018	0.7258 $\pm$ 0.0031
	PNN	0.7567 $\pm$ 0.0018	0.7301 $\pm$ 0.0033	0.7567 $\pm$ 0.0018	0.7331 $\pm$ 0.0034
	LMkNN	0.7609 $\pm$ 0.0015	0.7482 $\pm$ 0.0024	0.7609 $\pm$ 0.0015	0.7487 $\pm$ 0.0037
	LMPNN	0.7609 $\pm$ 0.0015	0.7482 $\pm$ 0.0024	0.7609 $\pm$ 0.0015	0.7487 $\pm$ 0.0037
	PAPNN	**0.7837 $\pm$ 0.0021**	**0.7614 $\pm$ 0.0040**	**0.7837 $\pm$ 0.0021**	**0.7507 $\pm$ 0.0014**
	FkNN	0.7085 $\pm$ 0.0023	0.7070 $\pm$ 0.0027	0.7085 $\pm$ 0.0023	0.7029 $\pm$ 0.0037
	EigenClass	0.7762 $\pm$ 0.0019	0.7528 $\pm$ 0.0032	0.7762 $\pm$ 0.0019	0.7550 $\pm$ 0.0022
	GMDkNN	0.7497 $\pm$ 0.0017	0.7294 $\pm$ 0.0034	0.7497 $\pm$ 0.0017	0.7343 $\pm$ 0.0026
	BMFkNN	0.6509 $\pm$ 0.0020	0.7027 $\pm$ 0.0034	0.6509 $\pm$ 0.0020	0.6674 $\pm$ 0.0047
	FPFS-kNN	0.7781 $\pm$ 0.0014	0.7571 $\pm$ 0.0026	0.7781 $\pm$ 0.0014	0.7573 $\pm$ 0.0031
	SVM	0.7620 $\pm$ 0.0013	0.5820 $\pm$ 0.0031	0.7620 $\pm$ 0.0013	0.6595 $\pm$ 0.0024
	RF	0.7459 $\pm$ 0.0014	0.7235 $\pm$ 0.0032	0.7459 $\pm$ 0.0014	0.7283 $\pm$ 0.0022
Bupa	kNN	0.6411 $\pm$ 0.0055	0.6530 $\pm$ 0.0067	0.6411 $\pm$ 0.0055	0.6360 $\pm$ 0.0011
	WkNN	0.6351 $\pm$ 0.0077	0.6501 $\pm$ 0.0102	0.6351 $\pm$ 0.0077	0.6312 $\pm$ 0.0069
	PNN	0.6479 $\pm$ 0.0066	0.6610 $\pm$ 0.0075	0.6479 $\pm$ 0.0066	0.6442 $\pm$ 0.0048
	LMkNN	0.6585 $\pm$ 0.0048	0.6683 $\pm$ 0.0060	0.6585 $\pm$ 0.0048	0.6454 $\pm$ 0.0041
	LMPNN	0.6585 $\pm$ 0.0048	0.6683 $\pm$ 0.0060	0.6585 $\pm$ 0.0048	0.6454 $\pm$ 0.0041
	PAPNN	0.6825 $\pm$ 0.0059	0.6858 $\pm$ 0.0064	0.6825 $\pm$ 0.0059	0.6764 $\pm$ 0.0072
	FkNN	0.6451 $\pm$ 0.0048	0.6618 $\pm$ 0.0061	0.6451 $\pm$ 0.0048	0.6389 $\pm$ 0.0019
	EigenClass	0.688 $\pm$ 0.0054	0.7061 $\pm$ 0.0056	0.688 $\pm$ 0.0054	0.6752 $\pm$ 0.0035
	GMDkNN	0.6349 $\pm$ 0.0058	0.6458 $\pm$ 0.0063	0.6349 $\pm$ 0.0058	0.6307 $\pm$ 0.0071
	BMFkNN	0.6190 $\pm$ 0.0075	0.6276 $\pm$ 0.0079	0.6190 $\pm$ 0.0075	0.6160 $\pm$ 0.0106
	FPFS-kNN	0.6030 $\pm$ 0.0061	0.6130 $\pm$ 0.0070	0.6030 $\pm$ 0.0061	0.5835 $\pm$ 0.0034
	SVM	0.5824 $\pm$ 0.0057	0.3709 $\pm$ 0.0254	0.5824 $\pm$ 0.0057	0.4339 $\pm$ 0.0097
	RF	**0.7481 $\pm$ 0.0025**	**0.7628 $\pm$ 0.0024**	**0.7481 $\pm$ 0.0025**	**0.7419 $\pm$ 0.0035**
Ionosphere	kNN	0.8426 $\pm$ 0.0036	0.8652 $\pm$ 0.0028	0.8426 $\pm$ 0.0036	0.8304 $\pm$ 0.0076
	WkNN	0.8487 $\pm$ 0.0032	0.8683 $\pm$ 0.0030	0.8487 $\pm$ 0.0032	0.8368 $\pm$ 0.0097
	PNN	0.8562 $\pm$ 0.0025	0.8738 $\pm$ 0.0024	0.8562 $\pm$ 0.0025	0.8448 $\pm$ 0.0072
	LMkNN	0.8958 $\pm$ 0.0023	0.9055 $\pm$ 0.0020	0.8958 $\pm$ 0.0023	0.8904 $\pm$ 0.0050
	LMPNN	0.8958 $\pm$ 0.0023	0.9055 $\pm$ 0.0020	0.8958 $\pm$ 0.0023	0.8904 $\pm$ 0.0050
	PAPNN	0.9215 $\pm$ 0.0018	0.9289 $\pm$ 0.0016	0.9215 $\pm$ 0.0018	0.9190 $\pm$ 0.0048
	FkNN	0.8443 $\pm$ 0.0034	0.8658 $\pm$ 0.0028	0.8443 $\pm$ 0.0034	0.8322 $\pm$ 0.0079
	EigenClass	0.9306 $\pm$ 0.0040	0.9316 $\pm$ 0.0045	0.9306 $\pm$ 0.0040	0.9300 $\pm$ 0.0124
	GMDkNN	0.8835 $\pm$ 0.0021	0.8936 $\pm$ 0.0024	0.8835 $\pm$ 0.0021	0.8778 $\pm$ 0.0058
	BMFkNN	0.8839 $\pm$ 0.0025	0.8923 $\pm$ 0.0033	0.8839 $\pm$ 0.0025	0.8773 $\pm$ 0.0048
	FPFS-kNN	0.6415 $\pm$ 0.0119	0.4234 $\pm$ 0.0157	0.6415 $\pm$ 0.0119	0.5073 $\pm$ 0.0152
	SVM	0.8830 $\pm$ 0.0022	0.8902 $\pm$ 0.0020	0.8830 $\pm$ 0.0022	0.8775 $\pm$ 0.0025
	RF	**0.9315 $\pm$ 0.0008**	**0.9345 $\pm$ 0.0007**	**0.9315 $\pm$ 0.0008**	**0.9313 $\pm$ 0.0008**
Pima-Indians	kNN	0.7375 $\pm$ 0.0019	0.7322 $\pm$ 0.0022	0.7375 $\pm$ 0.0019	0.7293 $\pm$ 0.0021
	WkNN	0.7327 $\pm$ 0.0032	0.7274 $\pm$ 0.0038	0.7327 $\pm$ 0.0032	0.7260 $\pm$ 0.0034
	PNN	0.7321 $\pm$ 0.0031	0.7276 $\pm$ 0.0039	0.7321 $\pm$ 0.0031	0.7246 $\pm$ 0.0028
	LMkNN	0.7417 $\pm$ 0.0020	0.7437 $\pm$ 0.0019	0.7417 $\pm$ 0.0020	0.7405 $\pm$ 0.0019
	LMPNN	0.7417 $\pm$ 0.0020	0.7437 $\pm$ 0.0019	0.7417 $\pm$ 0.0020	0.7405 $\pm$ 0.0019
	PAPNN	0.7473 $\pm$ 0.0033	0.7433 $\pm$ 0.0035	0.7473 $\pm$ 0.0033	0.7412 $\pm$ 0.0038
	FkNN	0.7359 $\pm$ 0.0019	0.7313 $\pm$ 0.0021	0.7359 $\pm$ 0.0019	0.7290 $\pm$ 0.0021
	EigenClass	0.7031 $\pm$ 0.0028	0.6957 $\pm$ 0.0044	0.7031 $\pm$ 0.0028	0.6721 $\pm$ 0.0018
	GMDkNN	0.7256 $\pm$ 0.0034	0.7224 $\pm$ 0.0038	0.7256 $\pm$ 0.0034	0.7218 $\pm$ 0.0029
	BMFkNN	0.7078 $\pm$ 0.0026	0.7041 $\pm$ 0.0029	0.7078 $\pm$ 0.0026	0.7031 $\pm$ 0.0025
	FPFS-kNN	0.7446 $\pm$ 0.0021	0.7406 $\pm$ 0.0025	0.7446 $\pm$ 0.0021	0.7364 $\pm$ 0.0029
	SVM	0.7708 $\pm$ 0.0018	0.7706 $\pm$ 0.0021	0.7708 $\pm$ 0.0018	0.7593 $\pm$ 0.0018
	RF	**0.7747 $\pm$ 0.0025**	**0.7731 $\pm$ 0.0027**	**0.7747 $\pm$ 0.0025**	**0.7696 $\pm$ 0.0027**
Parkinsons	kNN	0.8877 $\pm$ 0.0036	0.8911 $\pm$ 0.0054	0.8877 $\pm$ 0.0036	0.8743 $\pm$ 0.0117
	WkNN	0.9355 $\pm$ 0.0011	0.9414 $\pm$ 0.0010	0.9355 $\pm$ 0.0011	0.9338 $\pm$ 0.0047
	PNN	0.9268 $\pm$ 0.0015	0.9341 $\pm$ 0.0012	0.9268 $\pm$ 0.0015	0.9244 $\pm$ 0.0097
	LMkNN	0.8974 $\pm$ 0.0037	0.9077 $\pm$ 0.0029	0.8974 $\pm$ 0.0037	0.8876 $\pm$ 0.0126
	LMPNN	0.8974 $\pm$ 0.0037	0.9077 $\pm$ 0.0029	0.8974 $\pm$ 0.0037	0.8876 $\pm$ 0.0126
	PAPNN	0.9356 $\pm$ 0.0026	0.9400 $\pm$ 0.0024	0.9356 $\pm$ 0.0026	0.9344 $\pm$ 0.0061
	FkNN	0.9340 $\pm$ 0.0017	0.9412 $\pm$ 0.0014	0.9340 $\pm$ 0.0017	0.9317 $\pm$ 0.0095
	EigenClass	0.8703 $\pm$ 0.0031	0.8782 $\pm$ 0.0031	0.8703 $\pm$ 0.0031	0.8639 $\pm$ 0.0102
	GMDkNN	**0.9526 $\pm$ 0.0011**	**0.9583 $\pm$ 0.0009**	**0.9526 $\pm$ 0.0011**	**0.9515 $\pm$ 0.0048**
	BMFkNN	0.9412 $\pm$ 0.0021	0.9487 $\pm$ 0.0016	0.9412 $\pm$ 0.0021	0.9416 $\pm$ 0.0063
	FPFS-kNN	0.8969 $\pm$ 0.0029	0.9096 $\pm$ 0.0023	0.8969 $\pm$ 0.0029	0.8848 $\pm$ 0.0121
	SVM	0.8715 $\pm$ 0.0054	0.8874 $\pm$ 0.0042	0.8715 $\pm$ 0.0054	0.8505 $\pm$ 0.0086
	RF	0.9026 $\pm$ 0.0027	0.9178 $\pm$ 0.0014	0.9026 $\pm$ 0.0027	0.8962 $\pm$ 0.0034
Hill	kNN	0.5305 $\pm$ 0.0020	0.5363 $\pm$ 0.0020	0.5305 $\pm$ 0.0020	0.5302 $\pm$ 0.0025
	WkNN	0.5748 $\pm$ 0.0013	0.5800 $\pm$ 0.0015	0.5748 $\pm$ 0.0013	0.5746 $\pm$ 0.0019
	PNN	0.5642 $\pm$ 0.0013	0.5701 $\pm$ 0.0015	0.5642 $\pm$ 0.0013	0.5641 $\pm$ 0.0016
	LMkNN	0.6074 $\pm$ 0.0037	0.6113 $\pm$ 0.0037	0.6074 $\pm$ 0.0037	0.6074 $\pm$ 0.0041
	LMPNN	0.6074 $\pm$ 0.0037	0.6113 $\pm$ 0.0037	0.6074 $\pm$ 0.0037	0.6074 $\pm$ 0.0041
	PAPNN	**0.9447 $\pm$ 0.0005**	**0.9457 $\pm$ 0.0004**	**0.9447 $\pm$ 0.0005**	**0.9448 $\pm$ 0.0005**
	FkNN	0.5453 $\pm$ 0.0019	0.5506 $\pm$ 0.0020	0.5453 $\pm$ 0.0019	0.5447 $\pm$ 0.0016
	EigenClass	0.5293 $\pm$ 0.0018	0.5354 $\pm$ 0.0018	0.5293 $\pm$ 0.0018	0.5289 $\pm$ 0.0015
	GMDkNN	0.6258 $\pm$ 0.0022	0.6296 $\pm$ 0.0024	0.6258 $\pm$ 0.0022	0.6257 $\pm$ 0.0024
	BMFkNN	0.6215 $\pm$ 0.0030	0.6240 $\pm$ 0.0032	0.6215 $\pm$ 0.0030	0.6209 $\pm$ 0.0034
	FPFS-kNN	0.4793 $\pm$ 0.0017	0.4858 $\pm$ 0.0016	0.4793 $\pm$ 0.0017	0.4785 $\pm$ 0.0010
	SVM	0.4933 $\pm$ 0.0015	0.6581 $\pm$ 0.0218	0.4933 $\pm$ 0.0015	0.3599 $\pm$ 0.0026
	RF	0.5569 $\pm$ 0.0030	0.5607 $\pm$ 0.0029	0.5569 $\pm$ 0.0030	0.5574 $\pm$ 0.0029
Haberman-survival	kNN	0.7184 $\pm$ 0.0059	0.6894 $\pm$ 0.0108	0.7184 $\pm$ 0.0059	0.6737 $\pm$ 0.0044
	WkNN	0.6886 $\pm$ 0.0026	0.6601 $\pm$ 0.0058	0.6886 $\pm$ 0.0026	0.6563 $\pm$ 0.0032
	PNN	0.6949 $\pm$ 0.0041	0.6450 $\pm$ 0.0068	0.6949 $\pm$ 0.0041	0.6481 $\pm$ 0.0014
	LMkNN	0.6973 $\pm$ 0.0058	0.7028 $\pm$ 0.0082	0.6973 $\pm$ 0.0058	0.6823 $\pm$ 0.0092
	LMPNN	0.6973 $\pm$ 0.0058	0.7028 $\pm$ 0.0082	0.6973 $\pm$ 0.0058	**0.6824 $\pm$ 0.0092**
	PAPNN	0.7134 $\pm$ 0.0081	0.6505 $\pm$ 0.0291	0.7134 $\pm$ 0.0081	0.6553 $\pm$ 0.0039
	FkNN	0.686 $\pm$ 0.0024	0.6652 $\pm$ 0.0044	0.686 $\pm$ 0.0024	0.6594 $\pm$ 0.0032
	EigenClass	0.7014 $\pm$ 0.0078	0.6525 $\pm$ 0.0187	0.7014 $\pm$ 0.0078	0.6456 $\pm$ 0.0024
	GMDkNN	0.6686 $\pm$ 0.0041	0.6670 $\pm$ 0.0075	0.6686 $\pm$ 0.0041	0.6501 $\pm$ 0.0046
	BMFkNN	0.6381 $\pm$ 0.0049	0.6615 $\pm$ 0.0080	0.6381 $\pm$ 0.0049	0.6425 $\pm$ 0.0077
	FPFS-kNN	0.7136 $\pm$ 0.0068	**0.7184 $\pm$ 0.0105**	0.7136 $\pm$ 0.0068	0.6840 $\pm$ 0.0061
	SVM	**0.7297 $\pm$ 0.0071**	0.5506 $\pm$ 0.0192	**0.7297 $\pm$ 0.0071**	0.6246 $\pm$ 0.0144
	RF	0.6801 $\pm$ 0.0054	0.6784 $\pm$ 0.0094	0.6801 $\pm$ 0.0054	0.6632 $\pm$ 0.0071
Musk-1	kNN	0.8072 $\pm$ 0.0032	0.8359 $\pm$ 0.0020	0.8072 $\pm$ 0.0032	0.8078 $\pm$ 0.0011
	WkNN	0.8477 $\pm$ 0.0031	0.8660 $\pm$ 0.0020	0.8477 $\pm$ 0.0031	0.8489 $\pm$ 0.0022
	PNN	0.8552 $\pm$ 0.0027	0.8738 $\pm$ 0.0018	0.8552 $\pm$ 0.0027	0.8562 $\pm$ 0.0024
	LMkNN	0.8725 $\pm$ 0.0014	0.8800 $\pm$ 0.0012	0.8725 $\pm$ 0.0014	0.8723 $\pm$ 0.0008
	LMPNN	0.8725 $\pm$ 0.0014	0.8800 $\pm$ 0.0012	0.8725 $\pm$ 0.0014	0.8723 $\pm$ 0.0008
	PAPNN	**0.9047 $\pm$ 0.0027**	**0.9098 $\pm$ 0.0022**	**0.9047 $\pm$ 0.0027**	**0.9053 $\pm$ 0.0026**
	FkNN	0.8323 $\pm$ 0.0036	0.8580 $\pm$ 0.0023	0.8323 $\pm$ 0.0036	0.8331 $\pm$ 0.0027
	EigenClass	0.7089 $\pm$ 0.0024	0.7798 $\pm$ 0.0025	0.7089 $\pm$ 0.0024	0.7023 $\pm$ 0.0016
	GMDkNN	0.8844 $\pm$ 0.0025	0.8906 $\pm$ 0.0020	0.8844 $\pm$ 0.0025	0.8852 $\pm$ 0.0020
	BMFkNN	0.8797 $\pm$ 0.0026	0.8890 $\pm$ 0.0019	0.8797 $\pm$ 0.0026	0.8805 $\pm$ 0.0024
	FPFS-kNN	0.8181 $\pm$ 0.0024	0.8255 $\pm$ 0.0021	0.8181 $\pm$ 0.0024	0.8187 $\pm$ 0.0010
	SVM	0.8339 $\pm$ 0.0008	0.8374 $\pm$ 0.0008	0.8339 $\pm$ 0.0008	0.8338 $\pm$ 0.0008
	RF	0.8908 $\pm$ 0.0015	0.8930 $\pm$ 0.0016	0.8908 $\pm$ 0.0015	0.8905 $\pm$ 0.0015
Sonar	kNN	0.7618 $\pm$ 0.0079	0.7783 $\pm$ 0.0082	0.7618 $\pm$ 0.0079	0.7558 $\pm$ 0.0184
	WkNN	0.8542 $\pm$ 0.0059	0.8672 $\pm$ 0.0045	0.8542 $\pm$ 0.0059	0.8533 $\pm$ 0.0039
	PNN	0.8606 $\pm$ 0.0038	0.8761 $\pm$ 0.0029	0.8606 $\pm$ 0.0038	0.8589 $\pm$ 0.0036
	LMkNN	0.8440 $\pm$ 0.0061	0.8625 $\pm$ 0.0051	0.8440 $\pm$ 0.0061	0.8437 $\pm$ 0.0062
	LMPNN	0.8440 $\pm$ 0.0061	0.8625 $\pm$ 0.0051	0.8440 $\pm$ 0.0061	0.8437 $\pm$ 0.0062
	PAPNN	**0.8964 $\pm$ 0.0020**	**0.9040 $\pm$ 0.0019**	**0.8964 $\pm$ 0.0020**	**0.8965 $\pm$ 0.0022**
	FkNN	0.8155 $\pm$ 0.0051	0.8341 $\pm$ 0.0053	0.8155 $\pm$ 0.0051	0.8112 $\pm$ 0.0105
	EigenClass	0.7524 $\pm$ 0.0084	0.8104 $\pm$ 0.0058	0.7524 $\pm$ 0.0084	0.7459 $\pm$ 0.0053
	GMDkNN	0.8826 $\pm$ 0.0041	0.8937 $\pm$ 0.0035	0.8826 $\pm$ 0.0041	0.8828 $\pm$ 0.0025
	BMFkNN	0.8773 $\pm$ 0.0029	0.8880 $\pm$ 0.0025	0.8773 $\pm$ 0.0029	0.8775 $\pm$ 0.0042
	FPFS-kNN	0.8391 $\pm$ 0.0043	0.8608 $\pm$ 0.0036	0.8391 $\pm$ 0.0043	0.8366 $\pm$ 0.0049
	SVM	0.7640 $\pm$ 0.0058	0.7912 $\pm$ 0.0060	0.7640 $\pm$ 0.0058	0.7631 $\pm$ 0.0057
	RF	0.8364 $\pm$ 0.0020	0.8549 $\pm$ 0.0016	0.8364 $\pm$ 0.0020	0.8350 $\pm$ 0.0020
Wine	kNN	0.9629 $\pm$ 0.0040	0.9731 $\pm$ 0.0023	0.9629 $\pm$ 0.0040	0.9630 $\pm$ 0.0011
	WkNN	0.9588 $\pm$ 0.0042	0.9710 $\pm$ 0.0020	0.9588 $\pm$ 0.0042	0.9587 $\pm$ 0.0019
	PNN	0.9644 $\pm$ 0.0036	0.9751 $\pm$ 0.0018	0.9644 $\pm$ 0.0036	0.9645 $\pm$ 0.0011
	LMkNN	0.9767 $\pm$ 0.0018	0.9815 $\pm$ 0.0012	0.9767 $\pm$ 0.0018	0.9769 $\pm$ 0.0005
	LMPNN	0.9767 $\pm$ 0.0018	0.9815 $\pm$ 0.0012	0.9767 $\pm$ 0.0018	0.9769 $\pm$ 0.0005
	PAPNN	**0.9844 $\pm$ 0.0009**	**0.9879 $\pm$ 0.0005**	**0.9844 $\pm$ 0.0009**	**0.9846 $\pm$ 0.0005**
	FkNN	0.9651 $\pm$ 0.0034	0.9756 $\pm$ 0.0017	0.9651 $\pm$ 0.0034	0.9654 $\pm$ 0.0011
	EigenClass	0.9711 $\pm$ 0.0011	0.9815 $\pm$ 0.0004	0.9711 $\pm$ 0.0011	0.9733 $\pm$ 0.0007
	GMDkNN	0.9733 $\pm$ 0.0025	0.9803 $\pm$ 0.0013	0.9733 $\pm$ 0.0025	0.9733 $\pm$ 0.0005
	BMFkNN	0.9643 $\pm$ 0.0025	0.9705 $\pm$ 0.0018	0.9643 $\pm$ 0.0025	0.9643 $\pm$ 0.0021
	FPFS-kNN	0.9521 $\pm$ 0.0024	0.9643 $\pm$ 0.0014	0.9521 $\pm$ 0.0024	0.9527 $\pm$ 0.0014
	SVM	0.9833 $\pm$ 0.0006	0.9882 $\pm$ 0.0003	0.9833 $\pm$ 0.0006	0.9843 $\pm$ 0.0005
	RF	0.9777 $\pm$ 0.0013	0.9813 $\pm$ 0.0011	0.9777 $\pm$ 0.0013	0.9785 $\pm$ 0.0013
Cardiotocography	kNN	0.7511 $\pm$ 0.0005	0.7528 $\pm$ 0.0006	0.7511 $\pm$ 0.0005	0.7384 $\pm$ 0.0007
	WkNN	0.7817 $\pm$ 0.0005	0.7833 $\pm$ 0.0006	0.7817 $\pm$ 0.0005	0.7756 $\pm$ 0.0014
	PNN	0.7913 $\pm$ 0.0004	0.7956 $\pm$ 0.0006	0.7913 $\pm$ 0.0004	0.7817 $\pm$ 0.0010
	LMkNN	0.7890 $\pm$ 0.0004	0.7969 $\pm$ 0.0006	0.7890 $\pm$ 0.0004	0.7822 $\pm$ 0.0012
	LMPNN	0.7890 $\pm$ 0.0004	0.7969 $\pm$ 0.0006	0.7890 $\pm$ 0.0004	0.7822 $\pm$ 0.0012
	PAPNN	0.8049 $\pm$ 0.0002	0.8143 $\pm$ 0.0002	0.8049 $\pm$ 0.0002	0.7987 $\pm$ 0.0011
	FkNN	0.7860 $\pm$ 0.0003	0.7905 $\pm$ 0.0006	0.7860 $\pm$ 0.0003	0.7782 $\pm$ 0.0013
	EigenClass	0.7285 $\pm$ 0.0005	0.7395 $\pm$ 0.0004	0.7285 $\pm$ 0.0005	0.7153 $\pm$ 0.0007
	GMDkNN	0.8057 $\pm$ 0.0004	0.8102 $\pm$ 0.0005	0.8057 $\pm$ 0.0004	0.8015 $\pm$ 0.0012
	BMFkNN	0.7883 $\pm$ 0.0005	0.7945 $\pm$ 0.0007	0.7883 $\pm$ 0.0005	0.7862 $\pm$ 0.0006
	FPFS-kNN	0.7996 $\pm$ 0.0004	0.8010 $\pm$ 0.0007	0.7996 $\pm$ 0.0004	0.7860 $\pm$ 0.0013
	SVM	0.7840 $\pm$ 0.0006	0.7742 $\pm$ 0.0007	0.7840 $\pm$ 0.0006	0.7637 $\pm$ 0.0006
	RF	**0.8763 $\pm$ 0.0002**	**0.8818 $\pm$ 0.0003**	**0.8763 $\pm$ 0.0002**	**0.8737 $\pm$ 0.0003**
QSAR	kNN	0.8522 $\pm$ 0.0011	0.8572 $\pm$ 0.0013	0.8522 $\pm$ 0.0011	0.8531 $\pm$ 0.0019
	WkNN	0.8628 $\pm$ 0.0009	0.8668 $\pm$ 0.0009	0.8628 $\pm$ 0.0009	0.8635 $\pm$ 0.0010
	PNN	0.8647 $\pm$ 0.0009	0.8678 $\pm$ 0.0009	0.8647 $\pm$ 0.0009	0.8653 $\pm$ 0.0016
	LMkNN	0.8610 $\pm$ 0.0007	0.8619 $\pm$ 0.0007	0.8610 $\pm$ 0.0007	0.8597 $\pm$ 0.0004
	LMPNN	0.8610 $\pm$ 0.0007	0.8619 $\pm$ 0.0007	0.8610 $\pm$ 0.0007	0.8597 $\pm$ 0.0004
	PAPNN	**0.8722 $\pm$ 0.0009**	**0.8734 $\pm$ 0.0010**	**0.8722 $\pm$ 0.0009**	**0.8708 $\pm$ 0.0009**
	FkNN	0.8549 $\pm$ 0.0009	0.8591 $\pm$ 0.0010	0.8549 $\pm$ 0.0009	0.8557 $\pm$ 0.0019
	EigenClass	0.8072 $\pm$ 0.0011	0.8241 $\pm$ 0.0015	0.8072 $\pm$ 0.0011	0.8107 $\pm$ 0.0027
	GMDkNN	0.8637 $\pm$ 0.0007	0.8650 $\pm$ 0.0008	0.8637 $\pm$ 0.0007	0.8635 $\pm$ 0.0013
	BMFkNN	0.8269 $\pm$ 0.0008	0.8302 $\pm$ 0.0008	0.8269 $\pm$ 0.0008	0.8271 $\pm$ 0.0019
	FPFS-kNN	0.8528 $\pm$ 0.0008	0.8559 $\pm$ 0.0009	0.8528 $\pm$ 0.0008	0.8526 $\pm$ 0.0008
	SVM	0.8531 $\pm$ 0.0005	0.8557 $\pm$ 0.0005	0.8531 $\pm$ 0.0005	0.8508 $\pm$ 0.0006
	RF	0.8702 $\pm$ 0.0007	0.8711 $\pm$ 0.0008	0.8702 $\pm$ 0.0007	0.8680 $\pm$ 0.0008
Band	kNN	0.6792 $\pm$ 0.0055	0.6874 $\pm$ 0.0052	0.6792 $\pm$ 0.0055	0.6632 $\pm$ 0.0037
	WkNN	0.7365 $\pm$ 0.0047	0.7548 $\pm$ 0.0051	0.7365 $\pm$ 0.0047	0.7313 $\pm$ 0.0032
	PNN	0.7408 $\pm$ 0.0044	0.7595 $\pm$ 0.0038	0.7408 $\pm$ 0.0044	0.7336 $\pm$ 0.0039
	LMkNN	0.7083 $\pm$ 0.0055	0.7220 $\pm$ 0.0052	0.7083 $\pm$ 0.0055	0.6980 $\pm$ 0.0031
	LMPNN	0.7083 $\pm$ 0.0055	0.7220 $\pm$ 0.0052	0.7083 $\pm$ 0.0055	0.6980 $\pm$ 0.0031
	PAPNN	**0.7426 $\pm$ 0.0029**	**0.7654 $\pm$ 0.0025**	**0.7426 $\pm$ 0.0029**	**0.7289 $\pm$ 0.0038**
	FkNN	0.7059 $\pm$ 0.0055	0.7192 $\pm$ 0.0049	0.7059 $\pm$ 0.0055	0.6919 $\pm$ 0.0018
	EigenClass	0.6806 $\pm$ 0.0093	0.6883 $\pm$ 0.0112	0.6806 $\pm$ 0.0093	0.6449 $\pm$ 0.0019
	GMDkNN	0.7416 $\pm$ 0.0050	0.7626 $\pm$ 0.0048	0.7416 $\pm$ 0.0050	0.7408 $\pm$ 0.0055
	BMFkNN	0.7078 $\pm$ 0.0071	0.7235 $\pm$ 0.0066	0.7078 $\pm$ 0.0071	0.7036 $\pm$ 0.0089
	FPFS-kNN	0.6711 $\pm$ 0.0066	0.6786 $\pm$ 0.0055	0.6711 $\pm$ 0.0066	0.6546 $\pm$ 0.0050
	SVM	0.6907 $\pm$ 0.0062	0.7092 $\pm$ 0.0076	0.6907 $\pm$ 0.0062	0.6488 $\pm$ 0.0083
	RF	0.7261 $\pm$ 0.0071	0.7426 $\pm$ 0.0081	0.7261 $\pm$ 0.0071	0.7208 $\pm$ 0.0057
Pima	kNN	0.7397 $\pm$ 0.0014	0.7379 $\pm$ 0.0014	0.7397 $\pm$ 0.0014	0.7327 $\pm$ 0.0014
	WkNN	0.7311 $\pm$ 0.0013	0.7293 $\pm$ 0.0011	0.7311 $\pm$ 0.0013	0.7247 $\pm$ 0.0013
	PNN	0.7336 $\pm$ 0.0012	0.7323 $\pm$ 0.0011	0.7336 $\pm$ 0.0012	0.7266 $\pm$ 0.0014
	LMkNN	0.7414 $\pm$ 0.0018	0.7479 $\pm$ 0.0016	0.7414 $\pm$ 0.0018	0.7412 $\pm$ 0.0035
	LMPNN	0.7414 $\pm$ 0.0018	0.7479 $\pm$ 0.0016	0.7414 $\pm$ 0.0018	0.7412 $\pm$ 0.0035
	PAPNN	0.7551 $\pm$ 0.0013	0.7568 $\pm$ 0.0009	0.7551 $\pm$ 0.0013	0.7480 $\pm$ 0.0021
	FkNN	0.7388 $\pm$ 0.0013	0.7381 $\pm$ 0.0012	0.7388 $\pm$ 0.0013	0.7332 $\pm$ 0.0013
	EigenClass	0.6994 $\pm$ 0.0024	0.6934 $\pm$ 0.0020	0.6994 $\pm$ 0.0024	0.6652 $\pm$ 0.0016
	GMDkNN	0.7264 $\pm$ 0.0016	0.7288 $\pm$ 0.0013	0.7264 $\pm$ 0.0016	0.7236 $\pm$ 0.0021
	BMFkNN	0.7143 $\pm$ 0.0014	0.7168 $\pm$ 0.0013	0.7143 $\pm$ 0.0014	0.7100 $\pm$ 0.0024
	FPFS-kNN	0.7410 $\pm$ 0.0012	0.7400 $\pm$ 0.0012	0.7410 $\pm$ 0.0012	0.7342 $\pm$ 0.0029
	SVM	0.7681 $\pm$ 0.0013	0.7675 $\pm$ 0.0016	0.7681 $\pm$ 0.0013	0.7577 $\pm$ 0.0016
	RF	**0.7655 $\pm$ 0.0017**	**0.7629 $\pm$ 0.0018**	**0.7655 $\pm$ 0.0017**	**0.7594 $\pm$ 0.0018**
Wine (keel)	kNN	0.9660 $\pm$ 0.0018	0.9730 $\pm$ 0.0011	0.9660 $\pm$ 0.0018	0.9664 $\pm$ 0.0009
	WkNN	0.9627 $\pm$ 0.0019	0.9706 $\pm$ 0.0011	0.9627 $\pm$ 0.0019	0.9627 $\pm$ 0.0014
	PNN	0.9680 $\pm$ 0.0014	0.9745 $\pm$ 0.0009	0.9680 $\pm$ 0.0014	0.9680 $\pm$ 0.0011
	LMkNN	0.9711 $\pm$ 0.0008	0.9769 $\pm$ 0.0005	0.9711 $\pm$ 0.0008	0.9715 $\pm$ 0.0003
	LMPNN	0.9711 $\pm$ 0.0008	0.9769 $\pm$ 0.0057	0.9711 $\pm$ 0.0008	0.9715 $\pm$ 0.0003
	PAPNN	**0.9895 $\pm$ 0.0007**	**0.9879 $\pm$ 0.0004**	**0.9895 $\pm$ 0.0007**	**0.9843 $\pm$ 0.0004**
	FkNN	0.9680 $\pm$ 0.0016	0.9746 $\pm$ 0.0010	0.9680 $\pm$ 0.0016	0.9684 $\pm$ 0.0005
	EigenClass	0.9695 $\pm$ 0.0014	0.9755 $\pm$ 0.0009	0.9695 $\pm$ 0.0014	0.9698 $\pm$ 0.0008
	GMDkNN	0.9710 $\pm$ 0.0011	0.9770 $\pm$ 0.0007	0.9710 $\pm$ 0.0011	0.9716 $\pm$ 0.0004
	BMFkNN	0.9679 $\pm$ 0.0014	0.9744 $\pm$ 0.0009	0.9679 $\pm$ 0.0014	0.9682 $\pm$ 0.0024
	FPFS-kNN	0.9567 $\pm$ 0.0035	0.9667 $\pm$ 0.0019	0.9567 $\pm$ 0.0035	0.9566 $\pm$ 0.0021
	SVM	0.9830 $\pm$ 0.0006	0.9872 $\pm$ 0.0003	0.9830 $\pm$ 0.0006	0.9836 $\pm$ 0.0006
	RF	0.9830 $\pm$ 0.0012	0.9874 $\pm$ 0.0007	0.9830 $\pm$ 0.0012	0.9833 $\pm$ 0.0012
Steel	kNN	0.7069 $\pm$ 0.0010	0.7140 $\pm$ 0.0010	0.7069 $\pm$ 0.0010	0.7044 $\pm$ 0.0019
	WkNN	0.7202 $\pm$ 0.0008	0.7245 $\pm$ 0.0008	0.7202 $\pm$ 0.0008	0.7184 $\pm$ 0.0013
	PNN	0.7209 $\pm$ 0.0010	0.7274 $\pm$ 0.0010	0.7209 $\pm$ 0.0010	0.7191 $\pm$ 0.0016
	LMkNN	0.7144 $\pm$ 0.0008	0.7272 $\pm$ 0.0008	0.7144 $\pm$ 0.00087	0.71205 $\pm$ 0.0038
	LMPNN	0.7144 $\pm$ 0.0008	0.7272 $\pm$ 0.0008	0.7144 $\pm$ 0.0008	0.7120 $\pm$ 0.0038
	PAPNN	0.7405 $\pm$ 0.0008	0.7467 $\pm$ 0.0007	0.7405 $\pm$ 0.0008	0.7382 $\pm$ 0.0024
	FkNN	0.7227 $\pm$ 0.0010	0.7279 $\pm$ 0.0010	0.7227 $\pm$ 0.0010	0.7205 $\pm$ 0.0017
	EigenClass	0.6982 $\pm$ 0.0011	0.7140 $\pm$ 0.0011	0.6982 $\pm$ 0.0011	0.6904 $\pm$ 0.0020
	GMDkNN	0.7318 $\pm$ 0.0008	0.7374 $\pm$ 0.0008	0.7318 $\pm$ 0.0008	0.7305 $\pm$ 0.0017
	BMFkNN	0.7144 $\pm$ 0.0009	0.7208 $\pm$ 0.0010	0.7144 $\pm$ 0.0009	0.7136 $\pm$ 0.0025
	FPFS-kNN	0.6972 $\pm$ 0.0009	0.7060 $\pm$ 0.0008	0.6972 $\pm$ 0.0009	0.6904 $\pm$ 0.0031
	SVM	0.6939 $\pm$ 0.0008	0.6870 $\pm$ 0.0010	0.6939 $\pm$ 0.0008	0.6835 $\pm$ 0.0009
	RF	**0.7815 $\pm$ 0.0003**	**0.7894 $\pm$ 0.0002**	**0.7815 $\pm$ 0.0003**	**0.7807 $\pm$ 0.0002**
Mammographic	kNN	0.7965 $\pm$ 0.0012	0.8027 $\pm$ 0.0011	0.7965 $\pm$ 0.0012	0.7960 $\pm$ 0.0018
	WkNN	0.7805 $\pm$ 0.0009	0.7859 $\pm$ 0.0010	0.7805 $\pm$ 0.0009	0.7802 $\pm$ 0.0006
	PNN	0.8012 $\pm$ 0.0007	0.8073 $\pm$ 0.0007	0.8012 $\pm$ 0.0007	0.8008 $\pm$ 0.0009
	LMkNN	0.7802 $\pm$ 0.0008	0.7867 $\pm$ 0.0008	0.7802 $\pm$ 0.0008	0.7798 $\pm$ 0.0008
	LMPNN	0.7802 $\pm$ 0.0008	0.7867 $\pm$ 0.0008	0.7802 $\pm$ 0.0008	0.7798 $\pm$ 0.0008
	PAPNN	**0.8043 $\pm$ 0.0010**	**0.8093 $\pm$ 0.0010**	**0.8043 $\pm$ 0.0010**	**0.8038 $\pm$ 0.0008**
	FkNN	0.7839 $\pm$ 0.0010	0.7890 $\pm$ 0.0011	0.7839 $\pm$ 0.0010	0.7835 $\pm$ 0.0008
	EigenClass	0.7978 $\pm$ 0.0007	0.8058 $\pm$ 0.0009	0.7978 $\pm$ 0.0007	0.79689 $\pm$ 0.0004
	GMDkNN	0.7799 $\pm$ 0.0006	0.7850 $\pm$ 0.0006	0.7799 $\pm$ 0.0006	0.7796 $\pm$ 0.0008
	BMFkNN	0.7091 $\pm$ 0.0016	0.7138 $\pm$ 0.0015	0.7091 $\pm$ 0.0016	0.7084 $\pm$ 0.0019
	FPFS-kNN	0.8023 $\pm$ 0.0009	0.8085 $\pm$ 0.0009	0.8023 $\pm$ 0.0009	0.8018 $\pm$ 0.0011
	SVM	0.7927 $\pm$ 0.0021	0.8033 $\pm$ 0.0020	0.7927 $\pm$ 0.0021	0.7919 $\pm$ 0.0021
	RF	0.7975 $\pm$ 0.0002	0.8032 $\pm$ 0.0003	0.7975 $\pm$ 0.0002	0.7971 $\pm$ 0.0002

**Note:**

The best classification results for each data set are shown in bold.

**Table 3:** The settings of the compared algorithms.

Methods	Settings
kNN	$k = 1,2,...,20$
WkNN	$k = 1,2,...,20$
PNN	$k = 1,2,...,20$
LMkNN	$k = 1,2,...,20$
LMPNN	$k = 1,2,...,20$
PAPNN	$k = 1,2,...,20$
FkNN	$k = 1,2,...,20$
EigenClass	$k = 1,2,...,20$
GMDkNN	$k = 1,2,...,20,p = - 2$
BMFkNN	$k = 1,2,...,20,p = 0.5,q = 3,m = 1.5$
FPFS-kNN	$k = 1,2,...,20,Pearson$
SVM	None
RF	None

Accuracy, precision, recall, and F1-score and their standard deviations (SD) (Memis, Enginoǧlu & Erkan, 2022a) are used to evaluate the algorithms. Note that the best classification results for each data set are shown in bold in Table 2. It can be observed that the proposed PAPNN method achieves better performance comparing with the other twelve classifiers on the whole.

To facilitate the interpretation of the results in Table 2, ranking numbers of the best results and a pairwise comparison of the ranking results are shown in Tables 4 and 5, respectively. Note that the best ranking results are shown in bold in Table 4. It can be seen from Table 4 that PAPNN outperforms the other algorithms for $19$ datasets. Besides, it is clear from Table 5 that PAPNN outperforms the other algorithms for at least $12$ datasets in every metric in the pairwise comparisons. The results above illustrate that the proposed PAPNN method is superior to other methods on the whole.

**Table 4:** Ranking number of the best results for all algorithms compared among each other.

Methods	Accuracy	Precision	Recall	F1-Score	Total rank
kNN	0	0	0	0	0
WkNN	0	0	0	0	0
PNN	0	0	0	0	0
LMkNN	0	0	0	0	0
LMPNN	0	0	0	1	1
PAPNN	**11**	**11**	**11**	**11**	**44**
FkNN	0	0	0	0	0
EigenClass	0	0	0	0	0
GMDkNN	1	1	1	1	1
BMFkNN	0	0	0	0	0
FPFS-kNN	0	1	0	0	1
SVM	1	0	1	0	0
RF	6	6	6	6	24

**Note:**

The best ranking results are shown in bold.

**Table 5:** Ranking number of the best results for two algorithms compared *vs*. each other.

Methods	Accuracy	Precision	Recall	F1-score
PAPNN *vs*. kNN	19	19	19	19
PAPNN *vs*. WkNN	19	17	19	18
PAPNN *vs*. PNN	19	19	19	19
PAPNN *vs*. LMkNN	19	17	19	18
PAPNN *vs*. LMPNN	19	17	19	18
PAPNN *vs*. FkNN	19	17	19	18
PAPNN *vs*. EigenClass	17	16	17	18
PAPNN *vs*. GMDkNN	17	16	18	17
PAPNN *vs*. BMFkNN	18	17	18	18
PAPNN *vs*. FPFS-kNN	18	17	18	18
PAPNN *vs*. SVM	16	17	16	17
PAPNN *vs*. RF	13	12	13	12

In order to enhance persuasiveness, a nonparametric statistical test called Friedman test (Demsar, 2006; Garcia & Herrera, 2008; Derrac et al., 2011) is carried out to compare the performance of classifiers. In the Friedman test, the best classifier is ranked as $1$, the second best rank $2$, and so on. In the case of ties, average ranks are assigned. Let $R_m^i$ be the rank of the $m$th of $n$ methods on the $i$th of $t$ data sets. The average rank of $m$th method is ${R_m} = {1 \over t}\sum\nolimits_{i = 1}^t {R_i^m}$. Under the null hypothesis of the Friedman test, all competing methods nearly have similar classification performance and so the ranks ${R_m}$ should be equal. The Friedman statistics is defined as follows:

(11) $$\chi_F^2 = {{12t} \over {n(n + 1)}}\left[ {\sum_m {R_m^2} - {{n{{(n + 1)}^2}} \over 4}} \right].$$

When $t \; > \; 10$ and $n \; > \; 5$, Friedman statistics is distributed according to $\chi_F^2$ with $n - 1$ degrees of freedom.

The average ranks of thirteen methods are listed in Table 6. From Table 6, It can be observed that the proposed PAPNN method achieves the best ranking. According to Eq. (11), the accuracy, precision, recall, and F1-score values of the Friedman test statistic are $\chi_F^2 = 72.954$, $\chi_F^2 = 73.070$, $\chi_F^2 = 72.954$, $\chi_F^2 = 76.120$, respectively. With $12(k - 1)$ degrees of freedom and the critical value for the Friedman test given for with $k = 13$ is $21.026$ at a significance level of $\alpha = 0.05$. It can be concluded that the accuracy ( $72.954\; \gt\; 21.026$), precision ( $73.070\; \gt\; 21.026$), recall ( $72.954\; \gt\; 21.026$), and F1-score ( $76.120\; \gt\; 21.026$) values of the studied methods are significantly different. Now that the null hypothesis is rejected, a *post-hoc* test can be proceeded. The Nemenyi test (Demsar, 2006; Memis, Enginoǧlu & Erkan, 2022a) is used to compare all classifiers with each other.

**Table 6:** The average ranks of thirteen methods using Friedman test on real-world data sets.

Methods	Accuracy	Precision	Recall	F1-score
kNN	9.31	9.5	9.36	9.42
WkNN	8.47	8.52	8.47	8.18
PNN	6.44	6.94	6.44	6.78
LMkNN	5.78	5.36	5.78	5.57
LMPNN	5.78	5.36	5.78	5.52
PAPNN	1.84	2.42	1.84	2.26
FkNN	8.55	8.26	8.55	8.21
EigenClass	9.26	9.39	9.26	9.73
GMDkNN	6.05	5.68	6.05	5.63
BMFkNN	8.68	8.10	8.68	8.15
FPFS-kNN	8.78	8.84	8.78	8.60
SVM	7.34	8.31	7.39	8.84
RF	4.55	4.21	4.55	4.05

**Figure 1:**
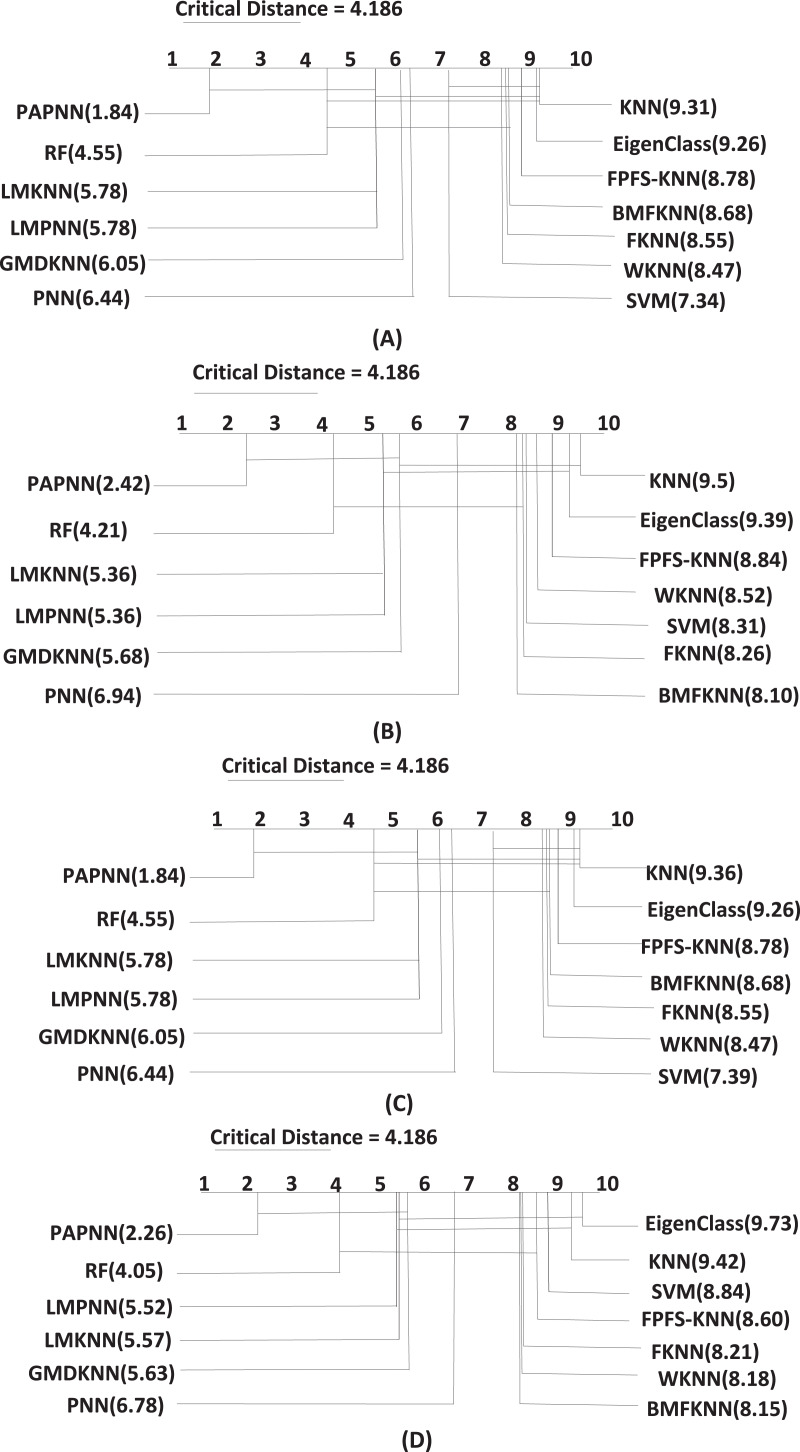
The critical diagrams for the four measures ((A) accuracy, (B) precision, (C) recall, (D) F1-score): the results from the Nemenyi *post hoc* test at $0.05$ significance level and average rank scores from Friedman test.

The critical value in the experiments with $k = 13$ and $\alpha = 0.05$ is $C{D_{0.05}} = 4.186$. As a result, the accuracy, precision, recall, and F1-score of the proposed PAPNN method is significantly different from kNN, WkNN, PNN, SVM, FPFS-kNN, FkNN, and BMFkNN methods, while it is not significantly different from RF, LMkNN, LMPNN, and GMDkNN method. Figure 1 presents the critical diagrams generated by the Nemenyi *post-hoc* test for the four performance measures.

### Experiments on high dimensional data sets

In this section, in order to further verify the performance of the proposed PAPNN, the experiments are conducted on three high dimensional data sets in terms of accuracy, precision, recall, and F1-score comparing to other kNN-based methods. The detailed information of the datasets can be found in the last three rows of Table 1. The values of $k$ are varied from $1$ to $20$ with a step of $1$. The comparative results are listed in Table 7. Note that the best classification result of thirteen methods on each high data set is highlighted in bold-face. It can be seen from Table 7 that the proposed PAPNN method achieves better classification performance than other nine methods on the whole.

**Table 7:** Comparative results for three high dimensional datasets.

Data	Methods	Accuracy $\pm$ SD	Precision $\pm$ SD	Recall $\pm$ SD	F1-score $\pm$ SD
AndrogenReceptor	kNN	0.8948 $\pm$ $1.45 \times {10^{ - 5}}$	0.8825 $\pm$ $2.81 \times {10^{ - 5}}$	0.8948 $\pm$ $1.45 \times {10^{ - 5}}$	0.8667 $\pm$ 0.0013
	WkNN	0.9051 $\pm$ $1.02 \times {10^{ - 5}}$	0.8979 $\pm$ $2.26 \times {10^{ - 5}}$	0.9051 $\pm$ $1.02 \times {10^{ - 5}}$	0.8827 $\pm$ 0.0009
	PNN	0.9044 $\pm$ $1.14 \times {10^{ - 5}}$	0.8931 $\pm$ $1.20 \times {10^{ - 5}}$	0.9044 $\pm$ $1.14 \times {10^{ - 5}}$	0.8827 $\pm$ 0.0009
	LMkNN	0.8816 $\pm$ $1.21 \times {10^{ - 5}}$	0.88203 $\pm$ $7.25 \times {10^{ - 6}}$	0.8816 $\pm$ $1.21 \times {10^{ - 5}}$	0.8817 $\pm$ 0.0001
	LMPNN	0.8846 $\pm$ $2.31 \times {10^{ - 5}}$	0.8821 $\pm$ $8.96 \times {10^{ - 6}}$	0.8846 $\pm$ $2.31 \times {10^{ - 5}}$	0.8832 $\pm$ $8.60 \times {10^{ - 5}}$
	PAPNN	**0.9132 $\pm$ $1.50 \times {10^{ - 6}}$**	**0.9122 $\pm$ $2.50 \times {10^{ - 6}}$**	**0.9132 $\pm$ $1.50 \times {10^{ - 6}}$**	**0.9121 $\pm$ $6.53 \times {10^{ - 5}}$**
	FkNN	0.8964 $\pm$ $9.36 \times {10^{ - 6}}$	0.8856 $\pm$ $1.51 \times {10^{ - 5}}$	0.8964 $\pm$ $9.36 \times {10^{ - 6}}$	0.8699 $\pm$ 0.0010
	GMDkNN	0.8950 $\pm$ $1.32 \times {10^{ - 5}}$	0.8847 $\pm$ $1.05 \times {10^{ - 5}}$	0.8950 $\pm$ $1.32 \times {10^{ - 5}}$	0.8885 $\pm$ $2.70 \times {10^{ - 5}}$
	BMFkNN	0.90737 $\pm$ $1.22 \times {10^{ - 5}}$	0.8951 $\pm$ $2.03 \times {10^{ - 5}}$	0.9073 $\pm$ $1.22 \times {10^{ - 5}}$	0.8926 $\pm$ 0.0003
	FPFS-kNN	0.8970 $\pm$ $4.42 \times {10^{ - 6}}$	0.8849 $\pm$ $8.60 \times {10^{ - 6}}$	0.8970 $\pm$ $4.42 \times {10^{ - 6}}$	0.8700 $\pm$ 0.0005
Semeion	kNN	0.9527 $\pm$ $6.55 \times {10^{ - 5}}$	0.9542 $\pm$ $4.25 \times {10^{ - 5}}$	0.9527 $\pm$ $6.55 \times {10^{ - 5}}$	0.9453 $\pm$ 0.0028
	WkNN	0.9463 $\pm$ $1.01 \times {10^{ - 5}}$	0.9448 $\pm$ $9.24 \times {10^{ - 6}}$	0.9463 $\pm$ $1.01 \times {10^{ - 5}}$	0.9405 $\pm$ 0.0003
	PNN	0.9485 $\pm$ $5.36 \times {10^{ - 6}}$	0.9483 $\pm$ $2.27 \times {10^{ - 6}}$	0.9485 $\pm$ $5.36 \times {10^{ - 6}}$	0.9427 $\pm$ 0.0003
	LMkNN	0.9604 $\pm$ $6.02 \times {10^{ - 5}}$	0.96156 $\pm$ $4.47 \times {10^{ - 5}}$	0.9604 $\pm$ $6.02 \times {10^{ - 5}}$	0.9557 $\pm$ 0.0024
	LMPNN	0.9672 $\pm$ $8.32 \times {10^{ - 6}}$	0.9674 $\pm$ $8.33 \times {10^{ - 6}}$	0.9672 $\pm$ $8.32 \times {10^{ - 6}}$	0.9645 $\pm$ 0.0003
	PAPNN	**0.9670 $\pm$ $1.73 \times {10^{ - 6}}$**	**0.9676 $\pm$ $1.41 \times {10^{ - 6}}$**	**0.9670 $\pm$ $1.73 \times {10^{ - 6}}$**	**0.9670 $\pm$ $6.71 \times {10^{ - 5}}$**
	FkNN	0.9533 $\pm$ $6.09 \times {10^{ - 5}}$	0.9549 $\pm$ $4.02 \times {10^{ - 5}}$	0.9533 $\pm$ $6.09 \times {10^{ - 5}}$	0.9462 $\pm$ 0.0026
	GMDkNN	0.9544 $\pm$ $2.34 \times {10^{ - 6}}$	0.9538 $\pm$ $5.98 \times {10^{ - 6}}$	0.9544 $\pm$ $2.34 \times {10^{ - 6}}$	0.9503 $\pm$ $3.43 \times {10^{ - 5}}$
	BMFkNN	0.94642 $\pm$ $1.72 \times {10^{ - 5}}$	0.9463 $\pm$ $1.12 \times {10^{ - 5}}$	0.9464 $\pm$ $1.72 \times {10^{ - 5}}$	0.9399 $\pm$ 0.0008
	FPFS-kNN	0.9621 $\pm$ $7.74 \times {10^{ - 6}}$	0.9610 $\pm$ $9.89 \times {10^{ - 6}}$	0.9621 $\pm$ $7.74 \times {10^{ - 6}}$	0.9592 $\pm$ 0.0001
CNAE-9	kNN	0.6279 $\pm$ 0.0060	0.7717 $\pm$ 0.0004	0.6279 $\pm$ 0.0060	0.6337 $\pm$ 0.0061
	WkNN	0.7406 $\pm$ $6.85 \times {10^{ - 5}}$	0.7827 $\pm$ $5.30 \times {10^{ - 5}}$	0.7406 $\pm$ $6.85 \times {10^{ - 5}}$	0.7402 $\pm$ $6.78 \times {10^{ - 5}}$
	PNN	0.7672 $\pm$ 0.0003	0.8178 $\pm$ 0.0003	0.7672 $\pm$ 0.0003	0.7660 $\pm$ 0.0003
	LMkNN	0.7977 $\pm$ 0.0003	0.82226 $\pm$ 0.0003	0.7977 $\pm$ 0.0003	0.7995 $\pm$ 0.0003
	LMPNN	0.8022 $\pm$ 0.0004	0.8191 $\pm$ 0.0004	0.8022 $\pm$ 0.0004	0.8031 $\pm$ 0.0004
	PAPNN	**0.8122 $\pm$ $8.54 \times {10^{ - 6}}$**	**0.8206 $\pm$ $7.67 \times {10^{ - 6}}$**	**0.8122 $\pm$ $8.54 \times {10^{ - 6}}$**	**0.8149 $\pm$ $8.10 \times {10^{ - 6}}$**
	FkNN	0.6554 $\pm$ 0.0034	0.7882 $\pm$ 0.0002	0.6554 $\pm$ 0.0034	0.6628 $\pm$ 0.0034
	GMDkNN	0.8084 $\pm$ 0.0008	0.8212 $\pm$ 0.0003	0.8084 $\pm$ 0.0008	0.8058 $\pm$ 0.0008
	BMFkNN	0.72875 $\pm$ 0.0002	0.7512 $\pm$ 0.0001	0.7287 $\pm$ 0.0002	0.7245 $\pm$ 0.0002
	FPFS-kNN	0.7967 $\pm$ 0.0003	0.81226 $\pm$ 0.0003	0.7957 $\pm$ 0.0003	0.7985 $\pm$ 0.0003

**Note:**

The best classification results for each data set are shown in bold.

### Running cost analysis

From the pseudo code of the PAPNN algorithm, the computational complexity is *O*( $Mn(n - 1)$) in terms of big *O* notation. Here, $n$ and *M* are the number of the training samples and of their attributes, respectively. PAPNN has higher complexity compared to the other aforesaid algorithms. Therefore, the PAPNN algorithm requires more time to complete its execution compared to other algorithms. The mean processing time data of all aforsaid algorithms on $19$ real datasets at twenty runs are listed in Tables 8–10. From Tables 8–10, it can be observed that PAPNN seems to operate significantly slower compared to other algorithms in the case of a relatively large number of sampling points and relatively high dimensionality. Despite this issue, PAPNN’s running time remains under $5$ s for thirteen out of the $19$ datasets.

**Table 8:** Average processing time for the real datasets (in seconds).

Methods	Vehicle	Balance	Blood	Bupa	Ionosphere	Pima-Indians	Parkinsons
kNN	0.0079	0.0062	0.0079	0.0042	0.0059	0.0076	0.0029
WkNN	0.0089	0.0069	0.0069	0.0038	0.0049	0.0079	0.0036
PNN	0.3248	0.0747	0.0868	0.0558	0.2461	0.1416	0.0927
LMkNN	0.3432	0.0754	0.0852	0.0560	0.2579	0.1477	0.1062
LMPNN	0.3850	0.0811	0.0907	0.0616	0.2846	0.1552	0.1313
PAPNN	6.1658	1.3197	2.6635	0.6329	3.3422	3.8297	0.7803
FkNN	0.0128	0.0089	0.0099	0.0059	0.0059	0.0109	0.0049
EigenClass	2.3763	0.6803	0.6891	0.3573	1.3862	0.8976	0.4995
GMDkNN	0.3936	0.0903	0.1080	0.0657	0.2970	0.1595	0.1303
BMFkNN	0.0416	0.0149	0.0169	0.0179	0.0643	0.0249	0.0538
FPFS-kNN	1.6477	0.3550	0.4280	0.2707	1.3852	0.9241	0.6141
SVM	0.3344	0.0579	0.0794	0.0388	0.0558	0.1136	0.0224
RF	2.9254	1.6681	1.8746	1.5895	2.5380	3.0331	1.9394

**Table 9:** Average processing time for the real datasets (in seconds).

Methods	Hill	Haberman-survival	Musk-1	Sonar	Wine	Cardiotocography	QSAR
kNN	0.0079	0.0062	0.0079	0.0042	0.0059	0.0076	0.0029
WkNN	0.0089	0.0069	0.0069	0.0038	0.0049	0.0079	0.0036
PNN	0.3248	0.0747	0.0868	0.0558	0.2461	0.1416	0.0927
LMkNN	0.3432	0.0754	0.0852	0.0560	0.2579	0.1477	0.1062
LMPNN	0.3850	0.0811	0.0907	0.0616	0.2846	0.1552	0.1313
PAPNN	199.6386	0.3864	35.6519	2.8254	0.2553	27.2365	46.0123
FkNN	0.0128	0.0089	0.0099	0.0059	0.0059	0.0109	0.0049
EigenClass	2.3763	0.6803	0.6891	0.3573	1.3862	0.8976	0.4995
GMDkNN	0.3936	0.0903	0.1080	0.0657	0.2970	0.1595	0.1303
BMFkNN	0.0416	0.0149	0.0169	0.0179	0.0643	0.0249	0.0538
FPFS-kNN	1.6477	0.3550	0.4280	0.2707	1.3852	0.9241	0.6141
SVM	0.3344	0.0579	0.0794	0.0388	0.0558	0.1136	0.0224
RF	2.9254	1.6681	1.8746	1.5895	2.5380	3.0331	1.9394

**Table 10:** Average processing time for the real datasets (in seconds).

Methods	Band	Pima	Wine (keel)	Mammographic	Steel
kNN	0.0056	0.0079	0.0038	0.0219	0.0087
WkNN	0.0045	0.0078	0.0040	0.0242	0.0069
PNN	0.1497	0.1597	0.0558	1.1043	0.1116
LMkNN	0.1568	0.1471	0.0630	1.1276	0.1120
LMPNN	0.1730	0.1568	0.0823	1.2106	0.1141
PAPNN	2.0690	4.2120	0.2546	34.710	3.2976
FkNN	0.0109	0.0170	0.0089	0.0388	0.0159
EigenClass	1.2020	1.4945	0.4132	8.1129	1.2795
GMDkNN	0.2523	0.2303	0.1296	1.7251	0.1576
BMFkNN	0.0390	0.0239	0.0358	0.0627	0.0197
FPFS-kNN	0.9335	0.9516	0.3944	9.0507	0.9944
SVM	0.0662	0.1055	0.0187	1.5800	0.1000
RF	2.1755	2.7420	1.3017	9.3959	2.3380

## Conclusion

In this article, a pre-averaged based pseudo nearest neighbor classifier (PAPNN) is proposed to improve the classification performance in the case of small-size training samples with existing outliers. The PAPNN rule, which is based on the average idea, can further reduce the negative impact of existing outliers to some degree. Classification performance of PAPNN is evaluated through an effective comparison with kNN, WkNN, PNN, LMkNN, LMPNN, FkNN, GMDkNN, BMFkNN, FPFS-kNN, EigenClass, SVM, and RF. The algorithms are trained and tested for $10$ runs using $10$-fold cross-validation over the nineteen real numerical data sets and three high dimensional data sets. The results are then evaluated using several well-known measures, such as accuracy, precision, recall, and F1-score. Statistical analysis and computational complexity are carried out to further check the accuracy and processing time of the algorithm by comparing with the other algorithms.

The experimental results and statistical analysis show that PAPNN has the best classification performance on the whole. This issue is the significant advantage of PAPNN. On the other hand, PAPNN’s disadvantage is that the processing time will become longer with the number of training set points increasing. This disadvantage may limit PAPNN to perform large-scale data classification on personal computers. To deal with this disadvantage, distributed computing or the hardware accelerator, such as graphics processing unit (GPU), can be employed to accelerate the classification process.

In general, the data at the class boundary is uncertain to some extent. The fuzzy algorithm can deal with this uncertainty to some degree, thus improving the accuracy and robustness of classification. Therefore, the future research should be focused on combining PAPNN with the fuzzy algorithm to improve the classification effect.

## Supplemental Information

10.7717/peerj-cs.2247/supp-1 Supplemental Information 1 The specific implementation code of the PAPNN method proposed in this article. The code mainly consists of the following parts: (1) Initialize and read the dataset into memory; (2) Normalize the data; (3) Implement the PAPNN algorithm program; (4) Implement the classification result statistics function.

10.7717/peerj-cs.2247/supp-2 Supplemental Information 2 Datasets involved in the experiments of this article.

10.7717/peerj-cs.2247/supp-3 Supplemental Information 3 Dataset path file. This file is required by the algorithm program proposed in this paper. Its specific content is the path on the disk where the experimental datasets are stored.

## Additional Information and Declarations

### Data Availability

The following information was supplied regarding data availability: Data and code are available in the Supplemental Files.
